# “Is the doctor God to punish me?!” An intersectional examination of disrespectful and abusive care during childbirth against single mothers in Tunisia

**DOI:** 10.1186/s12978-017-0290-9

**Published:** 2017-03-04

**Authors:** Nada Amroussia, Alison Hernandez, Carmen Vives-Cases, Isabel Goicolea

**Affiliations:** 1 Epidemiology and Global Health, Department of Public Health and Clinical Medicine, Umeå University, 901 87 Umeå, Sweden; 2 Department of Community Nursing, Preventive Medicine and Public Health and History of Science Alicante University, Alicante, Spain; 3 CIBER of Epidemiology and Public Health, Barcelona, Spain

**Keywords:** Tunisia, Childbirth, Single mothers, Maternal health, Abusive care, Disrespectful care, Intersectionality, Qualitative thematic analysis

## Abstract

**Background:**

Disrespectful and abusive treatment during childbirth is a violation of women’s right to dignified, respectful healthcare throughout pregnancy and childbirth. Although reports point out that marginalized groups in society such as single mothers are particularly vulnerable to abusive and disrespectful care, there is a lack of in-depth research exploring single mothers’ encounters at the maternal healthcare facilities, especially in Tunisia. In Tunisia, single mothers are particularly vulnerable due to their social stigmatization and socio-economic marginalization. This study examines the self-perceptions and childbirth experiences of single mothers at the public healthcare facilities in Tunisia.

**Methods:**

This study follows a qualitative design. Eleven single mothers were interviewed in regard to their experiences with maternal healthcare services and their perceptions of the attitudes of the health workers towards them. The interviews also addressed the barriers faced by the participants in accessing adequate maternal healthcare services, and their self-perceptions as single mothers. The data were analyzed using an inductive thematic approach guided by the feminist intersectional approach. Emergent codes were grouped into three final themes.

**Results:**

Three themes emerged during the data analysis: 1) Experiencing disrespect and abuse, 2) Perceptions of regret and shame attributed to being a single mother, and 3) The triad of vulnerability: stigma, social challenges, and health system challenges.

The study highlights that the childbirth experiences of single mothers are shaped by intersectional factors that go beyond the health system. Gender plays a major role in constructing these experiences while intersecting with other social structures. The participants had experienced disrespectful and discriminatory practices and even violence when they sought maternal healthcare services at the public healthcare facilities in Tunisia. Those experiences reflect not only the poor quality of maternal health services but also how health system practices translate the stigma culturally associated with single motherhood in this setting. Social stigma did not only affect how single mothers were treated during the childbirth, but also how they perceived themselves and how they perceived their care.

**Conclusion:**

Ensuring women’s right to dignified, respectful healthcare during childbirth requires tackling the underlying causes of social inequalities leading to women’s marginalization and discrimination.

**Electronic supplementary material:**

The online version of this article (doi:10.1186/s12978-017-0290-9) contains supplementary material, which is available to authorized users.

## Plain English summary

Abusive and disrespectful care during childbirth is a human rights violation as it violates women’s right to dignified, respectful healthcare throughout pregnancy and childbirth. Marginalized groups of women in society such as single mothers are particularly vulnerable to disrespectful and abusive care. The aim of this study is to examine the self-perceptions and childbirth experiences of single mothers at the public healthcare facilities in Tunisia.

Eleven single mothers were interviewed in regard to their experiences with maternal healthcare services and their perceptions of the attitudes of the health workers towards them. The interviews also addressed the barriers faced by the participants in accessing adequate maternal healthcare services, and their self-perceptions as single mothers.

This study shows that the participants (single mothers) experienced discriminatory and abusive practices when they sought maternal healthcare services at the public healthcare facilities in Tunisia. These experiences reflect not only the low quality of the healthcare services, but also how health system translates in its practices the discrimination and stigma culturally associated with single motherhood in this setting. Social discrimination and stigma did not only affect how single mothers were treated during the childbirth, but also how they perceived themselves and how they perceived their care. However, some signs of resistance existed.

## Background

Abusive and disrespectful care during childbirth represents a human rights violation as it violates women’s right to dignified, respectful healthcare throughout pregnancy and childbirth [1]. It is also considered a form of violence against women linked to the persistence of gender inequality in society [2]. Disrespectful and abusive treatment includes individual disrespect and abuse due to health workers’ behaviors such as verbal and physical violence, and structural disrespect and abuse due to systematic failures such as lack of hygiene in the maternity wards [3]. Abusive and disrespectful care has been linked to multiple adverse health outcomes including distress, poor self-rated health, posttraumatic stress disorder and sleeping disorders [4, 5]. It is also associated with maternal mortality and morbidity [4, 6].

While skilled facility-based delivery is considered an important factor in reducing maternal mortality in low and middle income countries, abusive care represents a significant barrier to use of maternal healthcare services as it reduces women’s trust in the health system [7]. A growing body of evidence indicates that abusive and disrespectful treatment during pregnancy and delivery in healthcare facilities is widespread in many low and middle income countries [8, 9], e.g., a recent study pointed out that the prevalence of non-dignified care in healthcare facilities in Kenya was 18% [10], other studies revealed that 15% of women who delivered in a referral hospital in Tanzania reported experiencing one or more forms of abusive and disrespectful care, and this proportion reached 78% among women who delivered in healthcare facilities in Ethiopia [11, 12]. Accordingly, the World Health Organization [1] called for multiplying research efforts to investigate care practices during childbirth as part of the commitment to ensure women’s right to dignified, respectful care throughout pregnancy and childbirth.

Abusive and disrespectful care disproportionally affects vulnerable groups of women in society including single mothers [8, 13]. Although reports [13] in Sierra Leone, Tanzania, and Vietnam, pointed out that single mothers were subjected to discriminatory attitudes from service providers when accessing maternal healthcare services, to our knowledge, there are no published studies exploring in depth single mothers’ experiences of abuse and disrespect during childbirth. Single mothers’ access to healthcare services is hampered by financial hardship [14], stigma related to taboos surrounding premarital sex, and discriminatory attitudes and practices of healthcare providers [13]. In Middle East and North Africa (MENA) region, tight restrictions on women’s sexuality and bodies contribute to a persistent rhetoric of shame and disgrace surrounding single mothers [15, 16]. In this context, social stigma, fear of being judged and feeling ashamed of seeking sexual and reproductive health services represent barriers to unmarried women’s access to and utilization of these services [17, 18]. Yet, there is a lack of actual knowledge about the experiences of maternal healthcare among unmarried women.

### Single mothers and maternal healthcare in Tunisia

Although the term “single mother(s)” may be used extensively in literature, in this study we used the term “single mother(s)” to refer to women who are not married at the time of the pregnancy and the baby’s birth.

In Tunisia, the organization “Santé Sud” stated that 1200 to 1600 babies are born outside of marriage every year [19, 20], while the total number of births in 2014 was 225 890 [21]. In 2014, an official survey based on a sample of 732 single mothers registered at the Ministry of Social Affairs described single mothers as young women, with low education level and a high unemployment rate [22]. For a long time, single mothers and their children were considered as invisible by the Tunisian legislation. It was only in 1998 that the law allowed children born outside of marriage to receive the father’s name. Single mothers receive limited economic and social support from the state due to the lack of social services targeting them. In response to this situation, different initiatives have been taken by civil society organizations to provide single mothers with financial, social and psychosocial services [20, 23].

Single mothers in Tunisia are considered one of the most marginalized groups in society and the issue of disrespect and abuse during childbirth is particularly relevant for them given their vulnerable status. Despite growing international attention to the problem of disrespectful and abusive care as an aspect of poor quality maternal healthcare services and a barrier to service utilization, no studies have yet been conducted to explore experiences of this phenomenon neither among Tunisian women in general, nor among single mothers in particular. In fact, few studies have investigated the quality of maternal healthcare services in Tunisia. These studies pointed out different forms of low quality maternal healthcare services such as underestimation of risk, inadequate follow-up during post partum and delay in appropriate treatment [24, 25]. Existing studies have highlighted social aspects of the stigma surrounding single motherhood and the discrimination that unmarried women face in accessing reproductive healthcare services [20, 23, 26, 27]. These findings point to the need for further research investigating the quality of maternal healthcare services in Tunisia with focus on single mothers’ childbirth experiences in this setting. To understand these experiences, it is also important to investigate how the treatment that single mothers received in delivery care is connected to broader societal and gender norms.

### Intersectional approach to single mothers’ self-perceptions and childbirth experiences

Women’s health experiences including sexual and reproductive health experiences, and their encounters with healthcare professionals are conventionally analyzed through a single-gender lens, while other social structures such as ethnicity and social class are considered as *“additive factors”*. This approach has been criticized as it does not fully capture the complexity of women’s health experiences [28–30]. The intersectional approach has been proposed as a better fit to understand and address health inequities, and to develop equity-based policies [28, 30]. Introduced by black feminist scholars in the 1980s, the intersectional approach was presented as a useful analytical framework to explore social phenomena. It focuses on the most marginalized groups in the society, while recognizing the diversity of their lived experiences [31, 32].

The intersectional approach assumes that the social locations of groups and individuals are determined by intersecting systems of power relations, where gender is one category of inequality, alongside class, age, ethnicity, gender identity, or others. The intersections of these categories create a complex web of social inequalities that are all experienced together by individuals. It is not possible to explore inequalities grounded in each category in isolation from each other, since that is not the way they are experienced by individuals. The intersection of these categories, creates positions of privilege and underprivilege, inclusion and exclusion, and these positions affect how individuals perceive themselves, and how they are perceived and treated by others. Health and healthcare experiences are also shaped by these axes of inequalities [29, 30].

In Tunisia, as elsewhere, the childbirth experiences of single mothers are shaped by a gender order that subordinates and controls women and their sexuality. However, gender relations do not operate in isolation from other social categories like social class. In fact, in this setting, single motherhood is mainly experienced by young women with low socio-economic status who are socially perceived as *morally deviant* and *promiscuous* [20, 23]. The underprivileged position of single mothers in Tunisia has to be understood within a web of converging patterns of subordination, built on gender but also on social class. Both categories interplay to restrict single mothers’ ability to enjoy their right to dignified, respectful healthcare during childbirth, and are manifested in the multiple and complex self-perceptions constructed by single mothers [33]. Self-perceptions refer to the evaluative judgments and attitudes that people use to describe themselves, and they are constructed by the individuals’ sense of esteem or agency. Self-perceptions are also influenced by the individuals’ understandings of how they are perceived and evaluated by others, and by how they are categorized in society (according to their gender, ethnicity, age, social class…) [34]. This can lead to multiple and intersectional *selves* developed by one person, and therefore, to a multifaceted self-perception constructed by this person [35].

The present study examines the self-perceptions and childbirth experiences of single mothers at the public healthcare facilities in Tunisia, and applies an intersectional approach to analyze their connection to the intertwined effects of gender relations and social class. The study had the following specific aims:

- Explore how single mothers perceived the attitudes of maternal healthcare providers towards them.
- Explore the challenges faced by single mothers to access adequate maternal healthcare services that ensure women’s right to dignified, respectful healthcare during childbirth.
- Explore the participants’ self-perceptions as single mothers.

## Methods

### Study setting

The study was conducted in Tunis district, Tunisia. Tunisia is a middle-income country located in North Africa and with a total population of 11 million. According to the constitution, Tunisia has a republic regime, Arabic is the official language and Islam is the official religion [36]. Gender inequality is persistent in the Tunisian society. In 2014, women represented 50.2% of the total population, while they represented only 25.88% of the total work force [36, 37]. The unemployment rate among women was twice that of men (22.6% vs 15.6%), and the same applies to the illiteracy rate (22.5% vs 12.5%) [37, 38].

Tunis district is formed by four subdistricts including the capital Tunis. The number of the population in this district is 2.504 million, with 92% living in an urban setting. In 2013, the unemployment rate in this region (16.9%) was slightly higher than the national rate [39]. Despite the availability and the accessibility of maternal healthcare services in this region, the maternal mortality rate is higher in Tunis district (50.8/100 000 live births) compared to the national rate. This is likely due to the poor quality of maternal healthcare services rather than lack of access [25].

This study was conducted in collaboration with two non-governmental organizations (NGOs), situated in Tunis district, Tunisia. One of these organizations targets only single mothers, while the other targets marginalized women living in urban settings including single mothers. Both organizations provide social, psychological and legal assistance services, housing services, and professional training for their target population [40].

### Study participants

Participants were contacted through the two NGOs, and were approached by the organizations’ employees. Women who were single mothers, had experienced the delivery at a public healthcare facility and were 18 years old or more at the moment of the interview were invited to participate in this study. Minor single mothers were excluded, and one participant withdrew from the study. Eleven participants receiving the organizations’ services agreed to participate in the present study. The participants’ age ranged from 19 years old to 43 years old, with seven participants younger than 30. Seven participants had basic educational level, three had secondary school education and one participant was illiterate. Six participants were unemployed and five were working in the informal sector. Only one participant did not have the Tunisian nationality (Algerian). Seven participants delivered at a University Teaching Hospital in the capital; while the other participants delivered at public healthcare facilities in different cities (3 different regional hospitals and one university teaching hospital in a coastal city). None of the participants was in relationship with the father of the child during the data collection period.

### Data collection

A semi-structured interview guide with open-ended questions was used. The interview guide was developed after reflecting on the knowledge and the experiences of the authors (see interview guide on Additional file 1). Four main topics were addressed in the interview thematic guide: the experiences of single mothers with maternal healthcare services in Tunisia, the participants’ perceptions of the maternal healthcare providers’ attitudes towards them, the barriers faced in accessing adequate maternal healthcare services, and the participants’ self-perceptions as single mothers. The interviews were carried out from December 2015 to January 2016 by the first author. The mean duration of the interviews was 30 min. The interviews were conducted in Arabic, the mother tongue of the interviewer and the participants. All the interviews were conducted face to face at the organizations’ offices; and were audio-taped. A notebook was used to register additional information about the participants, as well as memos and comments.

### Data analysis

The interviews were transcribed verbatim in Arabic and translated into English by the first author. This process contributed in enhancing familiarity with the data as transcripts were read repeatedly during the transcription and translation processes. The translation also contributed in facilitating the involvement of one of the co-authors in the analysis. The transcripts were analyzed using an inductive thematic analysis approach following Braun and Clarke [41]. The transcripts were coded line per line, following an inductive approach with the use of emergent codes. Afterwards emergent codes with similar ideas were grouped together. Codes and groups were further examined to look for thematic patterns in the data. Notes were used for clarification, i.e., using the comments and the background information to better understand the participants’ accounts. Five thematic patterns emerged during this process. The analysis was refined by introducing intersectional theory with focus on the intersection between gender relations and social class. The five thematic patterns were reviewed, and condensed into three final themes. The development and the refinement of the final themes entailed oscillating between an inductive approach and a deductive approach in order to ensure linking the data to the theoretical framework used i.e., the intersectional approach. This process involved constant back and forth movements from the codes, the groups and the preliminary thematic patterns to the theoretical framework [41].

## Results

Three themes emerged during the data analysis process: “Experiencing disrespect and abuse”, “Perceptions of regret and shame associated with being a single mother”, “The triad of vulnerability: stigma, social challenges, and health system challenges”.

### Experiencing disrespect and abuse

This theme describes the relations between single mothers and maternal healthcare professionals during childbirth. The participants recalled diverse experiences of abuse and disrespectful treatment during childbirth.

Participants mentioned that they felt neglected, ignored, and disrespected by the maternal healthcare providers who, for example, never asked for their consent before performing medical procedures. The participants often complained about suffering from hunger, cold and risking post-delivery complications as a result of the neglect.

*“After the delivery, I stayed the whole night in the cold. I used a hospital’s blanket to cover my son because he also stayed naked for the whole night(…) And, I was feeling hungry. Imagine… they gave me only a very cold soup.” (Sahar).*

Participants described how they felt judged by the health workers who openly blamed them for having a baby outside of marriage. Discriminatory practices experienced by the participants ranged from being treated differently from other women to denying access to postnatal healthcare services.

*“They didn’t ask me to come back…I saw that they told the other women to come back in prefixed dates, but they didn’t do the same for me. I was not completely healed.... They told all women to come back except me.” (Ferdaws).*

Participants also reported encountering different forms of psychological and verbal violence, including being insulted, scolded, and subjected to humiliation and threats from the healthcare providers who also violated their privacy. Some participants described being subjected to physical violence during the delivery. They mentioned being beaten on the hips, slapped on the face and having finger marks on their bodies.

*“The doctor was rude when she was making the sutures. She was shouting at me and screaming…She was beating me on my hips…Her attitude was not normal.” (Sawsen).*

Violence in its most severe form was experienced by the participant Mariem, who accused the healthcare professionals of committing a criminal act and stealing one of her baby twins after the delivery. The participant described how just after the birth of her twins, she was anesthetized by a nurse, whom she accused of disappearing after taking the child. She also mentioned that the hospital, where she delivered, ignored her claims. Throughout the interview, the participant described her fruitless efforts to find her son, her despair and her sadness.

*“I wish I can find my son…I wish that my son will come back to me…Every time I see a little boy, I remember my son.” (The participant started crying) (Mariem).*

Feeling targeted by mistreatment because of their status as single mothers was frequently mentioned by the participants. However, participants also described that abusive and disrespectful practices were common and normalized in the hospital, and affected women regardless of their marital status. These different perceptions are captured by the quotes below.

*“When the nurses and interns asked me about the father’s name…I explained my situation and I told them that I was a single mother… and the way they treated me changed…A nurse started screaming and shouting at me.” (Marwa).*

*“It wasn’t because of my status as single mother. They treated all women badly. The midwives became…I don’t know how to say it…maybe they got used to see women delivering.” (Sahar).*

Participants described how abuse made them feel offended, unsafe, powerless and desperate. The interviews also included instances where participants questioned health workers’ abuse and described refusing to be stigmatized, ignored or mistreated.

“*Was she there to punish me for my mistake?!!…Is the doctor God to punish me?!!… she is not God to judge people!!” (Sawsen).*

*“When the nurse started shouting at me…I told her: "that’s it, I will leave!” (Marwa).*

Although the experiences described were mainly negative, participants were also able to mention a few occasion when they felt well treated and satisfied with the maternal healthcare services.

*“At the moment of the delivery, I felt the contractions…It was very painful. The doctors asked me not to move…There were three doctors with me…and they told me to relax. (..) They treated me well…” (Kawthar).*

### Perceptions of regret and shame attributed to being a single mother

This theme describes the participants’ self-perceptions and their reflections about their overall experiences as single mothers. Participants considered their pregnancies outside of marriage as a *“mistake”*. The word *“mistake”* was repeated in all the interviews. Participants expressed feelings of regret, shame and guilt for making the *“mistake”* of being a single mother, and also tried to justify themselves based on lacking awareness or feeling distressed.

*“I made a mistake…and I had my son. My son was born outside of marriage “Haram”…“ (The participant started crying) (Amani).*

According to the participants, being a single mother is a burden that they have to cope with. Participants used expressions related to pain and suffering to describe their experiences as new single mothers, such as *“tough experience*”, “*painful experience”,* “*I suffered a lot*”, and *“I felt down”.*

Despite the predominant experiences of regret and shame reflected in their accounts, participants perceived being single mother as a challenge that allowed them to prove themselves to the society. They pointed out that single motherhood entailed also challenging the social norms and the social stigma, and they stressed the importance of being strong to face these challenges. Participants mentioned that in order to keep their children, they had to overcome the pressure from their families, from some health workers or the social services who encouraged them to have an abortion or to give their children in adoption. It is worth noting that according to the article 214 of the Tunisian Penal Code [42], women have the right to safe abortion during the first 12 weeks of pregnancy and exceptionally after this period if the pregnancy might compromise woman’s mental or physical health or if there is a risk of serious disability for the baby.

*“I don’t know…I think I was strong.. I insisted to keep my son and I challenged all the people and…I had to challenge my brothers, my family…my dad (RIP)…Yet, I was the only girl in the family, I should had made them proud as they said.” (Amira).*

*“The single mother cannot move forward, unless she ignores what people are saying…If she wants to move forward, she had to put society’s prejudices behind her…” (Sawsen).*

### The triad of vulnerability: stigma, social challenges, and health system challenges

Participants’ accounts of their childbirth experiences at public healthcare facilities and their self-perceptions as single mothers also illuminated socio-economic and cultural factors shaping their experiences, including stigma, marginalization and health system challenges. Together these factors contributed to the unique vulnerability of the participants as single mothers.

During the interviews, the participants reflected on how their families, the health workers, and the society in general referred to single mothers as “*mischievous woman”*, *“bad woman”*, *“without moral”*. The concept of “*Haram”*, which means forbidden by the religion, was also mentioned by the participants to describe the stigmatization that they had faced in the hospital or in the society as a whole. According to the participants, the bad image of single mothers did not only include being morally wrong but also being incapable of making decisions and assuming responsibilities as a mother. A participant described how she was suspected of abandoning her child by the social worker who did not consider her as a trustworthy mother.

*“Actually, I met the social worker… I explained my situation to her and she asked me to give her my I.D so I can return and take the baby. I left the I.D for two weeks, (while I was looking for housing)…Then, the social assistant said she was afraid that I will abandon the child in the future or do something bad to him…” (Sahar).*

The participants recalled also how they were rejected by their family, their friends, their partners and the society in general. They mentioned suffering from loneliness and isolation, and despair because of this rejection. As mentioned below by one of the participants, rejection is a common reaction of parents towards their daughters’ *“mistake”*.

*“If woman makes a mistake once, she has to pay it for the rest of her life… she will pay it…Her family will reject her.” (Ferdaws).*

Participants described experiencing socio-economic marginalization not only because of being single mothers, but also because they had basic education and came from poor families. Socio-economic marginalization was further reinforced during pregnancy or after childbirth. The majority of the participants mentioned how they struggled to afford necessities, how they lived in poverty and how homelessness was part of their experience as single mothers.

*“I was thinking about how can I take care of my baby…I didn’t know about organizations’ activities as I do now…I wasn’t aware of anything…I wasn’t in the same situation as now…I was worried about where I was going to live with her.” (Sawsen).*

*“I suffered a lot and I couldn’t give up on my daughter. I didn’t have an income and I couldn’t pay the rent…so I left the house and I had to stay with my sick baby in the hospital.” (The baby was suffering from a fetal distress and was hospitalized for a short period) (Farah).*

The participants also recounted how they assumed the whole responsibility of taking care of their children alone in absence of their partners. Some participants mentioned that the role of their partners was restricted to recognizing the baby by giving the child the father’s name. Other mentioned that they were completely abandoned by their partners before or after delivery. Few of them mentioned that they were accompanied by their partners during childbirth.

Participants complained about the poor quality of healthcare services in the hospital where they delivered: dirty facilities, poor quality of the food, lacking equipment, sharing beds in the maternal health department, bad quality of the sutures, a long waiting time and inappropriate episiotomy, were among the negative experiences they mentioned. Corrupt practices were also stated.

*“The guard was taking money from all people. I had to pay to even receive visits from my mother or my sister.” (Amani).*

Participants brought up the heavy workload of healthcare professionals. They partly justified their mistreatment during childbirth as being due to this heavy workload.

*“I can find excuses for the bad attitude of the one who assisted me in the delivery and for the doctor. I delivered by night…so maybe they had many women to assist in the delivery.” (Ferdaws).*

## Discussion

This study provides insight into single mothers’ different perceptions of discrimination and abuse when they sought maternal healthcare services at the public healthcare facilities in Tunisia. These experiences might reflect not only the bad quality of maternal healthcare services, but also how health system’s practices translate the social stigmatization surrounding single motherhood to the clinical encounters. Stigma along with other social difficulties contributed to a negative self-perception among the participants linked with being a single mother.

Abusive practices experienced by women during childbirth in the present study align with the different forms of abuse highlighted in previous studies conducted in low and middle income countries including neglect, discriminatory practices and physical and verbal violence [6, 8, 9, 13, 43–47]. Some of the aspects of low quality maternal care brought up by the participants in this study, such as inadequate episiotomies and dirty facilities, were also outlined in previous studies performed in low and middle income countries [45, 46]. Our study adds to this literature by investigating the phenomenon of abusive and disrespectful care among a marginalized group in the society and by connecting this phenomenon to a broader socio-cultural context using the intersectional approach [6, 8, 9, 13, 43–47].

To the best of our knowledge, this is the first health research paper from Africa to explicitly apply *intersectionality* to the study of women’s experiences of childbirth. In a recently published paper Larson et al. [48], called for more applications of the intersectional approach to health systems research in low and middle income countries. This intersectional approach might allow for new perspectives in understanding health systems’ deficiencies by considering the different social stratifiers of childbearing women and health workers as interlocked and mutually constructed.

### Single mothers’ social locations as underprivileged

In this study, the participants’ experiences and self-perceptions cannot only be explained by being single mothers. The participants have multiple identities: they are women, poor, low educated and single mothers. According to the intersectional approach, the effects of these factors are not additive but multiplicative, intertwined and directly affect the lived experiences of individuals [32]. As seen in this study, becoming a single mother might be associated to socio-economic marginalization, while at the same time single motherhood can further reinforce marginalization through the loss of social support and the burden of childbearing alone.

The negative symbolic image of single mothers in Tunisia is constructed through the patriarchal gender order that values women’s virginity and prohibits extra-marital sexual relationships [23]. Socio-cultural norms connected to religious beliefs play a determinant role in shaping this symbolic image through the concept of *“Haram”* frequently mentioned by the participants when they referred to their pregnancies outside of marriage. The symbolic image of single mothers was mirrored in the social stigmatization experienced by the participants. Stigma seems to also be embedded in the public institutions, and reflected in the emergence of a paternalistic approach in dealing with single mothers as mirrored in the social workers’ attitudes towards some participants. Stigma might have contributed to reinforcing the marginalization of single mothers as stigma can engender the loss of status for the stigmatized person. With lower status in the society’s hierarchy, the stigmatized person can experience many forms of inequalities and disadvantage including inequalities in access to socio-economic opportunities, education, and even in interactions with people [49].

### Disrespect and abuse faced by single mothers during childbirth

In Tunisia, there are no clearly written punitive policies against single mothers at the public healthcare facilities. Nevertheless, health workers’ abusive and discriminatory practices against single mothers were mentioned frequently by the participants, suggesting that these practices are a systematic issue, rather than any individual’s or group of individuals’ behaviors.

Most of the participants in this study stressed being targeted by health workers’ abuse because of their marital status. However, disrespectful and abusive care was also perceived as a common practice at the maternity wards and was linked to some deficiencies in the healthcare system. These nuanced perceptions of disrespectful and abusive care as well as the multiple identities exhibited by the study participants suggest diverse explanations for the participants’ negative childbirth experiences.

Violence, discriminatory and moralistic attitudes faced by the participants might be repressive practices used by the maternal healthcare providers to discipline single mothers from a moral perspective. Moral prejudices have been identified as one of the drivers of abusive care during childbirth, especially in low and middle income countries [8, 9]. Thereby, health workers are “*social actors”* contributing to sustain the ruling social and moral norms in a given society [47].

The abusive practices described by the participants in this study can be explained by unequal power relations between the women and the maternal healthcare providers [8], where gender as well as socio-economic status play an important role. Power relations are not only reflected in the moral prejudices towards women but also in the process of medicalization of childbirth itself, which disempowers women during the delivery and restricts their agency [50, 51]. During this process, violence and neglect can be used by the maternal healthcare providers, placed in an authority position, to punish certain women’s attitudes considered a menace to their authority such as not adhering to their instructions [2, 45]. Some of the rough attitudes can even be integrated in the obstetric training which can lead to the *normalization* of abuse [8, 52].

The unequal power relations might be also explained by the privileged position occupied by the health workers regarding access to education and social status compared to the participants. Women’s social class can affect healthcare providers’ attitudes. Poor women and lower-educated women, such as the ones in our study, may be subjected to abusive treatment during childbirth more often than wealthier and better-educated women [8, 50]. This might be relevant not only to the study participants but also to single mothers in general in this setting as according to the national survey conducted in 2014, 55.9% of single mothers had basic education and 21.1% had secondary education, only 1.8% of them had university education [22].

Taking into account the intersectional approach, we argue that the participants had experienced these different forms of power relations simultaneously. The single mother’s social location as underprivileged contributed largely in framing these unequal power relations. However, not all the childbirth experiences that participants recounted were of abuse or moral judgments. A few positive experiences were also described such as being assisted during the delivery by health workers whom were perceived as caring and supportive. These experiences indicate signs of resistance from certain maternal healthcare providers who contested the dominant social discourse of discrimination against single mothers. Resistance to the prevailing power relations also emerged from the participants themselves who tried sometimes to overcome their vulnerability in front of the health workers.

The practices of maternal healthcare providers are complex phenomena determined not only by the health workers’ characteristics and beliefs but also by factors at the organizational level of the health system [9]. Poor working conditions, heavy work load, and shortage in financial resources and equipment can lead to the demoralization and the dissatisfaction of health workers [8, 9]. Some of these factors such as heavy work load and inadequate equipment were brought up by the participants in the present study. The dissatisfaction of healthcare providers can affect their attitudes and contribute to aggravate the abusive care experienced by women at the healthcare facilities [8, 53]. Accountability mechanisms within the health system, for example, those that provide women with channels for registering complaints, might contribute to decreasing the risk of disrespect and abuse during facility-based childbirth [8].

### The social construction of the single mothers’ self-perceptions

Women’s self-perceptions as mothers are commonly generated from their ideal about motherhood and their real life experiences as mothers. While the ideals about motherhood are strongly stamped by the gender relations in a given society, the daily experiences of mothering depend on multiple factors including gender, religion and socio-economic status. Based on these factors, motherhood can be empowering or disempowering [54, 55].

In this study, the participants’ self-perceptions are marked by the negative image of single mothers in the society. Stigma and discrimination can affect people in different ways including internalizing stigma [56]. The internalization of stigma might explain some aspects of the participants’ self-perceptions such as guilt and shame. The acceptance of the negative stereotypes by the stigmatized people can also reduce their ability to resist their discrimination [49]. This might explain the limited ability of the participants to challenge the discriminatory practices of the maternal healthcare providers. The participants’ feelings of guilt might also be explained by their sense of failure to comply with the normative image of motherhood in Tunisia, an image that only exists within marriage [23]. These feelings of failure were reflected in the numerous repetitions of the word *“mistake”* in the participants’ discourses while describing their pregnancies outside of marriage. Our study also shows that the participants construct their self-perceptions as single mothers in isolation from their partners evoking an *invisibility* of men in most of their accounts.

However, participants not only expressed feeling of guilt and shame, but also a positive feeling of strength. Motherhood can be considered a “*creative project”* that encompasses defying the traditional assumptions about mothering [57]*.* In this sense, the participants’ choice to keep their children despite the difficulties encountered can be considered a form of challenging the hegemonic ideals about motherhood in the society.

### Methodological considerations

#### Measures to ensure the trustworthiness of the study

Several measures were taken in the present study to ensure trustworthiness [58]*. Triangulation* of researchers from different disciplines and with different levels of familiarity with the setting was used to enhance the credibility of the study by involving two of the authors in the different stages of designing the study protocol and analyzing the data. This allowed the combination of a cultural insider perspective together with an outsider perspective which adds to the trustworthiness of the study. To strengthen the transferability of this study, a detailed description of the context of the study was provided. To enhance dependability, the study adopted an emergent design throughout the research process which contributed in making single mothers’ voices more visible. To strengthen the confirmability, an inductive approach was used to develop the codes which entailed putting the pre-understanding of the phenomena studied *“between brackets”* [59]. The quotations were also used as a way to enhance confirmability. Collecting notes during the interviews was used to maximize both dependability and confirmability.

#### Study limitations

This study has limitations. We included all women regardless of the date of the delivery and the duration of the interviews because the participants provided a detailed description of their childbirth experiences. Nevertheless, this could have affected the way they recalled their experiences. The sample size was also restricted due to the limited data collection period and the sensitivity of the topic i.e., single mothers’ childbirth experiences that did not allow for recruiting a larger number of participants. However, in the final interviews similar issues and ideas started to emerge and we considered that the gathered information was enough to answer our research questions. As part of the data analysis process, the transcripts were translated from Arabic to English. Some information and nuances might have been lost in the translation process. Moreover, it was not possible logistically to share the findings of this study with the participants in order to revise and confirm our interpretation. This restricted our ability to follow-up on the different experiences recalled by the participants. Nevertheless, other measures were taken to ensure the trustworthiness of the present study as clarified above.

Only women who benefited from the organizations’ services were involved in the study. While the participants shared some profile characteristics with single mothers presented in the national survey for example having a low level of education and being unemployed, we do not claim that our study captured the multiple realities of single mothers’ experiences in Tunisia. The participants in this study might be those most in need of these organizations’ services; or might be better off compared to other single mothers in Tunisia because they benefited from the organizations’ support. Being supported by the organizations might have influenced the signs of resistance expressed by the participants in this study. Nevertheless, as single mothers are considered a stigmatized and hard to reach group in Tunisia, recruiting the participants through NGOs was considered more feasible for a first study exploring the childbirth experiences of single mothers in this setting. This also allowed to ensure receiving support from these NGOs in case some issues arose during the interviews. Moreover, in the present study, we wanted to focus on single mothers’ experiences as a group, rather than focusing on the intracategorical differences between these women. This choice led to portraying single mothers as a homogenous group, while this might not be the case. Furthermore, it seemed that the participants in this study perceived that some health workers were more empathic towards them compared to others. It would have been interesting to examine in-depth whether health workers’ attitudes differ according to their professional roles. Further research is needed to probe the different aspects of a variety of single mothers’ childbirth experiences and to compare and contrast these experiences.

Our study captured single mothers’ experiences and perceptions of the quality of care they received during childbirth. While it is important to highlight the perceptions of the participants as services users, we cannot claim for sure that they were subjected to abuse because they were single mothers. The participants’ perceptions might be the combination of different features of vulnerability which cannot be disentangled, and implying the use of *intersectionality*. Further research is needed to explore the practices of maternal healthcare providers towards different groups of women in Tunisia. Both qualitative and quantitative research is needed to assess the burden of abusive care and to gain insight into different experiences of women during facility-based childbirth. Examining maternal healthcare providers’ perceptions and experiences is equally important to be able to address the issue of disrespectful and abusive care during childbirth.

While our study applied *intersectionality* to examine single mothers’ experiences, it would also be interesting to explore the partners’ (men) experiences grounded in the same socio-economic and cultural context. Scholars [60] suggested applying intersectionality not only to understand women’s underprivilege encounters but also to gain an insight on how men’s experiences are constructed.

## Conclusions

This study showed how that the single mothers have experienced diverse forms of abuse and disrespectful treatment during childbirth at the public healthcare facilities in Tunisia. These negative childbirth experiences are constructed through the intersecting conditions of social stigma, socio-economic marginalization and a weak public health system. They reflected not only the poor quality of maternal health services but also how social stigma surrounding single motherhood can be embedded in the health system, and reproduced in the abusive and discriminatory practices of the maternal healthcare providers. Stigma and socio-economic marginalization also affected how the participants perceived themselves as single mothers and how they perceived their care.

Abusive care during the childbirth is a public health issue that requires greater efforts to generate evidence of the different forms of rights’ violations experienced by women at the healthcare facilities and guide enactment of solutions in policy and practice. Both women as service users and health workers as service providers should be involved in designing and implementing these policies with special focus on marginalized groups.

The study also highlighted some signs of resistance that involved some maternal healthcare providers, and some participants who tried to contest abusive care. Perhaps these signs of resistance could be strengthened both by empowering the women, and by empowering the healthcare workers, decreasing their patients loads, and improving their working conditions. Strengthening accountability mechanisms and encouraging women to report violations with special focus on the most vulnerable and marginalized groups might also decrease abusive practices.

Ensuring women’s right to dignified care also requires tackling the underlying causes of women’s marginalization and disempowerment. In Tunisia, the alarming situation of single mothers requires urgent measures from the state to support them, which include access to education, training and economic opportunities, and the end of social and institutional discrimination.

## Additional file

Additional file 1: Interview guide. (DOCX 12.1 kb)

## Abbreviations

MENA: Middle East and North Africa
NGOs: Non-governmental organizations
