# Supplementary material for: “Is the doctor God to punish me?!” An intersectional examination of disrespectful and abusive care during childbirth against single mothers in Tunisia
Source: Reprod Health. 2017 Mar 4;14:32. doi: 10.1186/s12978-017-0290-9 (PMC5336668; doi:10.1186/s12978-017-0290-9)
Supplement: Additional file 1: — Interview guide. (DOCX 12.1 kb) [file 12978_2017_290_MOESM1_ESM.docx]

**Interview guide**

The interview will start by letting the participant presenting herself.

1. Could you please present yourself?
2. When did you have your last child? Where did you have your last child?
3. Could you please tell me about your delivery experience?
4. Did you benefit from pre-natal care services? Could you please describe your first contact with the maternal care services?
5. Did you benefit from post-natal care services? How do you think about these services?
6. Did you face any difficulty in accessing to maternal health services? Could you please tell us more about these difficulties?
7. What do you think about the health workers' attitudes?
8. How do you explain the health workers' attitudes?
9. How do you feel about your overall experience in contact with maternal health services?
10. How you do you feel about being single mother in Tunisia?
